# The COVID-19 pandemic and war

**DOI:** 10.1177/1403494821993732

**Published:** 2021-02-21

**Authors:** Alexi Gugushvili, Martin Mckee

**Affiliations:** 1 University of Oslo, Norway; 2 London School of Hygiene and Tropical Medicine, UK

**Keywords:** COVID-19, war, global health

## Abstract

Could there be a symbiotic relationship between COVID-19 and conflict? On the one hand, circumstances associated with armed conflicts may give rise to greater spread of the virus, while, on the other hand, the COVID-19 pandemic may create conditions for violence through heightened xenophobia and nationalism or may change the dynamics of existing conflicts. We illustrate this with the example of war in the South Caucasus, one of the hot spots of the pandemic. Elsewhere, COVID-19 may have reduced the intensity of conflicts in some places, but it also may have contributed to anti-government protests and communal violence. We call for greater emphasis on traditional public health measures in unstable settings coupled with actions to hasten the peaceful resolution of ongoing conflicts.

The global response to the current pandemic has, albeit controversially, been framed as ‘the war against COVID-19’ [1]. Media attention has focused on the rising death toll, now over 2.36 million. The year 2020 was also marked by an estimated 119,010 deaths in armed confrontations such as wars, communal and state violence and riots, with many more people left with life-changing injuries [2]. At the beginning of the pandemic, UN Secretary-General António Guterres appealed for an immediate global ceasefire to enable the world to confront ‘a common enemy’ but his plea went largely unheeded [3]. In this commentary, we argue that there is a bidirectional relationship between COVID-19 and conflicts: on the one hand, circumstances associated with wars may facilitate pandemic spread; on the other hand, COVID-19 has already heightened xenophobia and nationalism, which in turn can encourage armed confrontations. In addition, a declining intensity of conflicts in 2020, with fewer war-related fatalities, was also associated with a large number of protests and violent attacks, some directly linked to the COVID-19 pandemic. Unquestionably, the combined effect of the virus and war is dire for population wellbeing, and for local and global social and health equity.

Wars and epidemics have a long and close history, going back at least to the well-documented Plague of Athens in the 5th century BCE [4]. A century ago, the influenza virus spread globally, facilitated by mass movement of military and civilian populations as the First World War drew to a close [5]. Although contemporary conflicts are on a much smaller scale, this is scant reassurance for communities affected in Syria, Yemen and many other places. The means by which viruses spread, as well as the barriers to mounting an effective response, are prominent during small-scale conflicts. The mobilisation of large numbers of soldiers and supporting personnel in 881 ongoing battles in December 2020, the forcible displacement of 80 million individuals in all parts of the world, and the concentration of hundreds of thousands of refugees in crowded camps with poor hygiene in the Middle East, East Africa and Southeast Asia [6] all are likely to be contributing to the COVID-19 pandemic.

The threat posed by COVID-19 can itself fuel conflict. We have already seen how it has encouraged nationalism and xenophobia [7, 8], with populist politicians blaming ‘others’, such as Trump’s use of the term ‘China virus’ coinciding with attacks on people of East Asian appearance [9]. History teaches us that these sentiments have often contributed to violence and conflict [10] with the competing nationalisms of the 1990s Yugoslav Wars providing a vivid example of this [11]. The COVID-19 pandemic may change the dynamics of pre-existing conflicts as epidemics play a major role in how political leaders are perceived [12]. Vulnerable social groups may turn against conventional forms of power, support radical political platforms and direct blame to parties with whom they have pre-existing frustrations [13]. Although shrinking economies may reduce states’ capacity to fund wars and support armed proxy groups, the COVID-19 pandemic can open doors for new domestic or interstate rivalries [14]. As some nations handle lockdowns, recessions and associated sociopolitical tensions better than others, they might miscalculate and become emboldened, raising the risk of renewed conflicts [15].

Of particular concern for the spread of coronavirus is how new or previously frozen conflicts have erupted in the midst of the pandemic. In the Caucasus, the setting for several conflicts in the aftermath of the collapse of the USSR, a longstanding dispute between Azerbaijan and Armenia erupted in September 2020. Both sides mobilised armed forces and engaged with heavy weapons from the outset. We do not suggest that there is a causal association between the pandemic and the initiation of the second Nagorno-Karabakh War between Azerbaijan and Armenia, as geopolitical factors are clearly playing a key role. Nonetheless, Armenia has been among the worst affected countries in the pandemic, with a cumulative total of 5680 cases per 100,000, and 1055 deaths per one million population [16]. The resurgent war, and eventually the Russian peace building mission on the ground, has been disastrous for the region and risked creating the conditions for a major outbreak of COVID-19. The dangerously overstretched healthcare systems in both countries, along with that in neighbouring Georgia, simultaneously dealt with one of the worst second waves of the COVID-19 pandemic and an influx of refugees and soldiers wounded during the war [17].

There is, however, some good news. Notwith-standing flare-ups such as that in the Caucasus and ongoing wars in Afghanistan, Somalia and Iraq, there is some evidence that the pandemic has reduced the intensity of some conflicts [18]. This view is supported by evidence from the Armed Conflict Location and Event Data Project, which reports a substantial reduction in both armed conflicts and deaths (Figure 1). The number of fatalities declined globally by more than a fifth compared to 2019 although not to the same extent everywhere; in South America, for instance, one of the pandemic’s hotspots, fatalities declined by only 8.6%.

**Figure 1.**
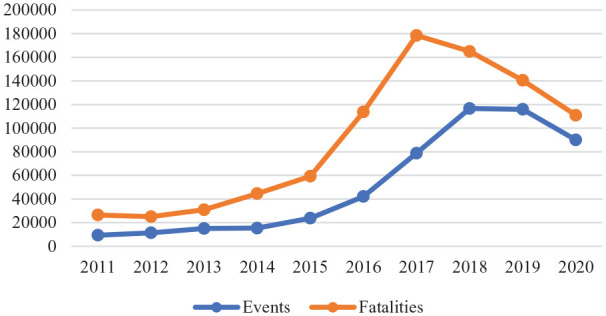
Armed conflicts around the world in 2011–2020. Armed conflicts count includes all episodes of battles, violence against civilians, explosions/remote violence and riots. Source: Armed Conflict Location and Event Data Project.

These findings should, however, be tempered by other evidence that, despite extensive lockdowns, the number of recorded protests worldwide has increased to more than 139,000 in 2020, that is 68.5% higher than in the previous year [2]. Part of this increase can be explained by specific developments around the world such as the global protests related to the death of George Floyd in police custody in the US, and the continuing pro-democracy protests in Hong Kong and Belarus. But there were also an estimated 33,247 protests directly related to COVID-19, such as demonstrations against regulations imposed in response to the pandemic, violent mobs attacking individuals due to fears of their alleged links to the coronavirus spread (e.g. Muslims in India, foreigners in some African countries), and the targeting of healthcare workers responding to the crisis [19]. These protests are an obvious marker of public discontent that can easily be exploited by powerful forces. Here, history again offers a warning. Those German municipalities that suffered most in the 1918 influenza outbreak were the ones that saw the greatest electoral gains for the Nazi Party a decade later [20]. The association between the spread of infections and armed conflicts can go in either direction depending on how the COVID-19 pandemic will affect political leaders’ legitimacy, the balance of power, domestic and interstate alliances and incentives for aggression.

The symbiotic relationship between conflict and infection calls on the global health community to demand action. Reducing the toll of avoidable death and disability will require a combination of traditional public health measures in a pandemic and policies that hasten peaceful resolution of major ongoing conflicts such as the Syrian Civil War, the secession conflict in Ukraine and the Mexican war on drugs. Yet the challenges are many, not least because of how many paramilitary groups have used the lockdowns during the pandemic to consolidate their influence in the resultant power vacuum [21]. As we gradually recover from the biggest public health crisis in generations, global leaders should consider including a peace-building component in the looming and immensely challenging global COVID-19 vaccination programme. Focusing vaccination efforts on countries severely affected by wars and conflicts could also be an effective strategy against the pandemic as these are areas where conditions for the continuous spread of the virus are particularly high.

## References

[1] Sabucedo JM Alzate M Hur D. COVID-19 and the metaphor of war (COVID-19 y la metáfora de la guerra). Rev Psicol Soc 2020;35:1–7.

[2] ACLED. The Armed Conflict Location & Event Data Project. 2020. https://acleddata.com/ (accessed 3 February 2021).

[3]] GuterresA. Transcript of the Secretary-General’s virtual press encounter on the appeal for global ceasefire. United Nations Secretary-General. https://www.un.org/sg/en/content/sg/press-encounter/2020-03-23/transcript-of-the-secretary-generals-virtual-press-encounter-the-appeal-for-global-ceasefire (2020, accessed 29 September 2020).

[4] Cooter R. Of war and epidemics: unnatural couplings, problematic conceptions. Soc Hist Med 2003;16:283–302. 14518485 10.1093/shm/16.2.283

[5] Humphries MO. Paths of infection: the First World War and the origins of the 1918 influenza pandemic. War Hist 2014;21:55–81.

[6] UNHCR. Global trends: forced displacment in 2019. Copenhagen. 2020. https://www.unhcr.org/5ee200e37.pdf (accessed 3 February 2021).

[7] Woods ET Schertzer R Greenfeld L , et al. COVID-19, nationalism, and the politics of crisis: a scholarly exchange. Nations Natl 2020;1–19. 10.1111/nana.12644 PMC7404753 32837223

[8]] GugushviliA KoltaiJ StucklerD , et al. Votes, populism, and pandemics. Int J Public Health 2020;65:721–722.3274068510.1007/s00038-020-01450-yPMC7394929

[9] McKee M Gugushvili A Koltai J , et al. Are populist leaders creating the conditions for the spread of COVID-19? Comment on “A scoping review of populist radical right parties’ influence on welfare policy and its implications for population health in Europe”. Int J Heal Policy Manag 2020;1–5. 10.34172/ijhpm.2020.124 PMC9056195 32668893

[10] Schrock-Jacobson G. The violent consequences of the nation: nationalism and the initiation of interstate war. J Conflict Resolut 2012;56:825–852.

[11] Pavković A. The fragmentation of Yugoslavia: Nationalism and war in the Balkans, 2nd ed. New York: St Martin’s Press, 2000.

[12] Roy M Moreau N Rousseau C , et al. Ebola and localized blame on social media: analysis of Twitter and Facebook conversations during the 2014–2015 Ebola epidemic. Cult Med Psychiatry 2020;44:56–79. 31214902 10.1007/s11013-019-09635-8 PMC7088957

[13] Gugushvili A. A population health perspective on the Trump administration, Brexit, and right-wing populism in Europe. Am J Public Health 2020;110:274–276. 32023103 10.2105/AJPH.2019.305535 PMC7002956

[14] Seaman RM (ed). Epidemics and war: The impact of disease on major conflicts in history. Santa Barbara: ABC-CLIO, 2018.

[15] Berman L Tischler J. After the calamity: unexpected effects of epidemics on war. RealClearDefense. 2020. https://www.realcleardefense.com/articles/2020/07/30/after_the_calamity_unexpected_effects_of_epidemics_on_war_115509.html (accessed 3 February 2021).

[16] John Hopkins University. Coronavirus Resource Center. https://coronavirus.jhu.edu/map.html (2020, accessed 5 October 2020).

[17] Hovhannisyan N Tsvetkova M. Armenia fights war with COVID-19 complicated by Nagorno-Karabakh conflict. Reuters. https://www.reuters.com/article/uk-armenia-azerbaijan-coronavirus/armenia-fights-war-with-covid-19-complicated-by-nagorno-karabakh-conflict-idUKKBN27Q1YT (2020, accessed 10 November 2020).

[18] *The Arab Weekly* . ISIS regrouping, exploiting COVID-19 disruptions, according to UN. 26 August 2020. https://thearabweekly.com/isis-regrouping-exploiting-covid-19-disruptions-according-un (accessed 3 February 2021).

[19] ACLED. Coronavirus-Related Events in the ACLED Dataset. https://acleddata.com/analysis/covid-19-disorder-tracker/ (2020, accessed 18 November 2020).

[20] Blickle K. Pandemics change cities: Municipal spending and voter extremism in Germany, 1918–1933. No. 921. New York: Federal Reserve Bank of New York, 2020.

[21] Grillo I. How Mexico’s Drug Cartels Are Profiting From the Pandemic. The New Youk Times, 2020. https://www.nytimes.com/2020/07/07/opinion/sunday/mexico-drug-cartels-coronavirus.html (accessed 3 February 2021).

